# A study of stromal riboflavin absorption in *ex vivo* porcine corneas using new and existing delivery protocols for corneal cross‐linking

**DOI:** 10.1111/aos.12884

**Published:** 2015-09-30

**Authors:** Sally Hayes, Siân R. Morgan, David P. O'Brart, Naomi O'Brart, Keith M. Meek

**Affiliations:** ^1^ School of Optometry and Vision SciencesCardiff UniversityCardiffUK; ^2^ Department of OphthalmologySt. Thomas’ HospitalLondonUK

**Keywords:** cornea, cross‐linking, iontophoresis, spectrophotometry

## Abstract

**Purpose:**

To indirectly measure stromal riboflavin penetration using commercially available riboflavin solutions and new and existing epithelium‐off, trans‐epithelial and iontophoresis‐assisted delivery protocols.

**Methods:**

Forty porcine eyes were divided into eight groups. Group 1: Ricrolin applied to the de‐epithelialised cornea for 30 min; Group 2: epithelium‐intact, no treatment; Groups 3–5: epithelium‐intact, 30‐min application of Ricrolin TE, Mediocross TE or ParaCel/Vibex, respectively. Group 6: epithelium‐intact, Ricrolin^+^ iontophoresis‐assisted delivery for 5 min; Group 7: epithelium‐intact, Ricrolin^+^ iontophoresis‐assisted delivery for 5 min with a 20‐min riboflavin soak; and Group 8: epithelium‐intact, Ricrolin^+^ iontophoresis‐assisted delivery for 5 min, 15‐min soak and another 5 min of iontophoresis. After a saline wash, light transmission spectra were obtained from each cornea, before and after epithelial removal.

**Results:**

Corneas in groups 1 and 8 showed a distinct riboflavin absorption peak between 400 and 520 nm. The optical density of the corneas in groups 3–7 did not differ significantly from that of the untreated corneas (group 2).

**Conclusions:**

A modification to the standard iontophoresis trans‐epithelial technique resulted in successful penetration of riboflavin into the stroma and appears to offer the most promise for epithelium‐on cross‐linking.

## Introduction

Riboflavin/ultraviolet A (UVA) 370 nm radiation corneal cross‐linking (CXL) has become increasingly popular as a means of stabilizing corneas that have been biomechanically weakened by disease or refractive surgery (Wollensak et al. 2003). The basis for this therapy lies in the knowledge that the incidence of naturally occurring cross‐links within the corneal macromolecules increases with age, resulting in progressive tissue stiffening (Elsheikh et al. 2007). Furthermore, keratoconus, a condition characterized by thinning and weakening of the cornea, rarely progresses beyond the age of 40 (Olivares Jimenez et al. 1997) and is less prevalent among patients with diabetes due to the acceleration of the glycation cross‐linking process (Mohammad et al. 2014). It was postulated that artificial cross‐linking using riboflavin and UVA 370 nm radiation could induce a similar stiffening effect, particularly in conditions such as keratoconus, where the collagen architecture within the corneal stroma is susceptible to enzymatic degradation (Kenney et al. 1994; Sawaguchi et al. 1994) and fibrillar slippage (Meek et al. 2005; Hayes et al. 2007). Clinical studies of CXL, with follow‐up times of up to 6 years, have shown it to be an effective means of halting keratoconus progression in over 90% of cases (Raiskup‐Wolf et al. 2008; O'Brart et al. 2013) with minimal reports of complications (Meek & Hayes 2013) and no change in corneal drug permeability following treatment (Tapeiner et al. 2014).

Despite being a relatively noninvasive and safe procedure, the standard CXL protocol requires removal of the central 7–9 mm of the corneal epithelium, prior to instillation of a hyperosmolar riboflavin solution and exposure to UVA irradiation (Wollensak et al. 2003). In an attempt to speed visual rehabilitation and reduce both the postoperative patient discomfort (Ghanem et al. 2013) and the risk of infection associated with epithelial debridement (Sharma et al. 2010), the concept of trans‐epithelial stromal riboflavin delivery has become increasingly popular and prompted research into alternative means of riboflavin delivery without the need for epithelial removal. However, the difficulty in delivering riboflavin across an intact corneal epithelium was evident in such research from the outset as riboflavin is a large hydrophilic molecule (molecular weight 340 Da) and the lipophilic epithelium has a decreasing permeability to molecules over 180 Da (Huang et al. 1989). Our previous spectrophotometry investigations into the effectiveness of complete epithelial removal versus partial epithelial disruption or the application of 20% alcohol or 1% tetracaine to loosen the epithelial tight junctions prior to the application of dextran‐enriched riboflavin solution showed that a sufficient and homogenous stromal uptake of riboflavin could only be achieved by complete removal of the epithelial barrier (Hayes et al. 2008; Samaras et al. 2009).

Recently, transepithelial riboflavin solutions have been developed which claim to facilitate the passage of riboflavin across the intact epithelium. The majority of these solutions are dextran free [with the exception of Ricrolin TE (Sooft Italia S.p.A., Montegiorgio, Italy)], on the basis that the high molecular weight of dextran is believed to inhibit the penetration of riboflavin across the epithelium (Huang et al. 1989; Hayes et al. 2008; Wollensak & Iomdina 2009). In the dextran‐free trans‐epithelial solutions, hydroxypropyl methycellulose (HPMC) is often used as a riboflavin vehicle and additional chemical agents, such as benzalkonium chloride (BAC), ethylenediamine tetra‐acetic acid (EDTA) and tometamol (Tris), are added to enhance the penetration of riboflavin through the corneal epithelium into the stroma(Ramselaar et al. 1988). The use of iontophoresis‐assisted riboflavin delivery, in which a low‐intensity electrical current is used to drive the negatively charged, water‐soluble riboflavin molecules through the corneal epithelium, is also being explored.

Although trans‐epithelial CXL undoubtedly offers patients a less invasive treatment than the standard epithelium‐off technique and thereby facilitates the treatment of paediatric and unco‐operative patients, as well those with very thin corneas, its effectiveness remains uncertain (Leccisotti & Islam 2010; Caporossi et al. 2012; Filippello et al. 2012; Bouheraoua et al. 2014). As riboflavin is essential to the CXL process, acting both as a photosensitizer for the production of oxygen free radicals to activate cross‐link formation and as an absorber of UVA radiation to prevent damage to internal ocular structures, the effectiveness of the therapy is dependent on adequate stromal riboflavin uptake.

In this study, we use spectrophotometry to indirectly measure and compare stromal riboflavin penetration in ex vivo porcine corneas following the standard (epithelium‐off) protocol, four clinically available trans‐epithelial riboflavin delivery protocols and two modified iontophoretic protocols.

## Methods

### Samples and treatments

Forty porcine eyes with intact corneal epitheliums were transported on ice from a local abattoir within 4 hr of death. On arrival, the corneas were randomly and equally divided into the eight treatment groups described below and summarized in Table 1:

**Table 1:** Summary of treatments and riboflavin composition

Group	Type and composition of riboflavin solution used	Epi on/off	Stromal delivery method	Treatment Abbreviation
1	Ricrolin: 0.1% riboflavin, 20% dextran, purified water	Off	Direct application	Epi‐off, Ricrolin
2	N/A	On	N/A	Epi‐on, No Tx
3	Ricrolin TE: 0.1% riboflavin, 15% dextran, Tris, EDTA, purified water	On	Trans‐epithelial	Epi‐on, Ricrolin TE
4	Mediocross TE: 0.25% riboflavin, 1.2% HPMC, 0.01% BACS, Pi‐water	On	Trans‐epithelial	Epi‐on, Mediocross TE
5	ParaCel: riboflavin 0.25%, HPMC, BACS, NaCl, EDTA, Tris. Vibex Xtra: 0.22% riboflavin, NaCl	On	Trans‐epithelial	Epi‐on, Paracel/Vibex
6	Ricrolin^+^: 0.1% riboflavin, Tris, EDTA, purified water	On	Trans‐epithelial/iontophoresis assisted	Epi‐on, Ricrolin^+^ and Ionto
7	Ricrolin^+^: 0.1% riboflavin, Tris, EDTA, purified water	On	Trans‐epithelial/iontophoresis assisted (with 20 min soak time)	Epi‐on, St Thomas’/Cardiff Ionto A
8	Ricrolin^+^: 0.1% riboflavin, Tris, EDTA, purified water	On	2 × trans‐epithelial/ionto‐phoresis assisted (with 15 min soak time)	Epi‐on, St Thomas’/Cardiff Ionto B

**Figure 1:**
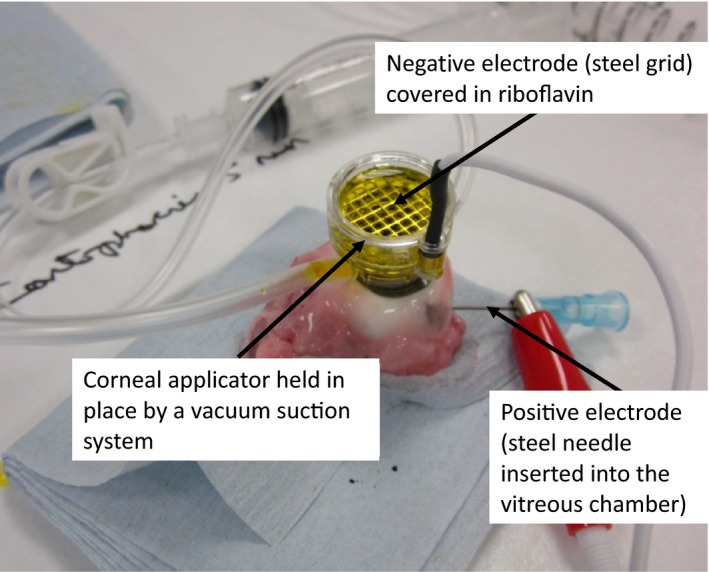
Iontophoresis riboflavin delivery system modified for use in ex vivo eyes.

1. Epithelium‐removed riboflavin delivery (epi‐off, Ricrolin): Following complete debridement of a central 10 mm area of corneal epithelium with a scalpel blade, riboflavin eye drops containing 0.1% riboflavin and 20% dextran T500 (Ricrolin, Sooft Italia S.p.A., Montegiorgio, Italy) were applied for 30 min.
2. Epithelium‐intact, untreated controls (Epi‐on, no Tx)
3. Ricrolin trans‐epithelial riboflavin delivery (Epi‐on, Ricrolin TE): Ricrolin TE (Sooft Italia S.p.A., Montegiorgio, Italy) was applied to the anterior surface of epithelium‐intact corneas for 30 min, followed by a 2‐min 0.9% saline rinse.
4. Mediocross trans‐epithelial riboflavin delivery (Epi‐on, Mediocross TE): Mediocross TE (Peschke GmbH, Waldshut‐Tiengen, Germany) was applied to the anterior surface of epithelium‐intact corneas for 30 min. This was followed by a 2 min 0.9% saline rinse.
5. Paracel/Vibex trans‐epithelial riboflavin delivery (Epi‐on, ParaCel/Vibex): ParaCel (Avedro, Waltham, MA, USA) was applied to the anterior surface of epithelium‐intact corneas for 4 min. This was followed by the application of Vibex Xtra (Avedro, Waltham, MA, USA) for a further 6 min and then a 2‐min 0.9% saline rinse.
6. Ricrolin^+^ trans‐epithelial, iontophoresis‐assisted riboflavin delivery (Epi‐on, Ricrolin^+^ and Ionto): Ricrolin^+^ ((Sooft Italia S.p.A., Montegiorgio, Italy) was delivered trans‐epithelially by means of 1 mA iontophoresis for 5 min (total charge of 300 mC). The iontophoresis delivery system was used to treat *ex vivo* eyes by connecting the return electrode to a needle inserted into the vitreous chamber; the negative electrode was a steel grid contained in a corneal well applicator which was adhered to the eye by means of a vacuum system (Fig. 1). The steel grid (negative electrode) was completely covered with riboflavin and the power generator set to the desired current and duration. The steel grid remained covered with riboflavin solution for the entire procedure. After treatment, the applicator was removed from the cornea and the cornea was rinsed with 0.9% saline solution for 2 min.
7. Modified Ricrolin^+^ trans‐epithelial, iontophoresis‐assisted riboflavin delivery (Epi‐on, St Thomas’/Cardiff Ionto A): Ricrolin^+^ was delivered trans‐epithelially by means of 1 mA iontophoresis for 5 min (total charge of 300 mC) and left *in situ* for a 20‐min soak time. Following treatment, the cornea was washed with 0.9% saline solution for 2 min.
8. Modified Ricrolin^+^ trans‐epithelial, iontophoresis‐assisted riboflavin delivery (Epi‐on, St Thomas’/Cardiff Ionto B): Ricrolin^+^ was delivered trans‐epithelially by means of 1 mA iontophoresis for 5 min and a 15‐min riboflavin soak, followed by 0.5 mA iontophoresis for 5 min (total charge of 450 mC). After treatment, the cornea was washed with 0.9% saline solution for 2 min.

Riboflavin solutions were administered by means of a suction ring (with vacuum syringe) designed for CXL and LASEK procedures which adhered to the corneal surface (e.Janarch srl, Como, Italy). Although the corneas in this study were not exposed to UVA, every attempt was made to mimic the clinical riboflavin application procedure. On the basis that riboflavin accumulation within the epithelium could cause UVA absorption and reduce the dosage of UVA to the stroma, a 2‐min post‐treatment saline rinse was performed on each cornea in groups 3–8 to remove riboflavin from the intact epithelium. It was not necessary to perform a saline rinse on corneas is group 1 (as the epithelium was removed) or group 2 (as no riboflavin was applied).

### Data collection

**Figure 2:**
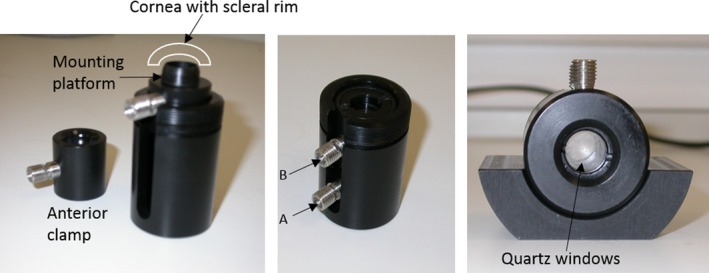
Sample holder used for spectrophotometry studies. After mounting the cornea on the sample platform, the anterior clamp is positioned to form a tight seal around the tissue. Silicon oil is then injected on either side of the cornea, first into the posterior chamber of the holder (A), to maintain the natural curvature of the cornea, and then into the anterior chamber (B).

Central corneal thickness measurements were recorded immediately following treatment using a Pachette2^™^ Ultrasonic Pachymeter (DGH Technology, Exton, PA, USA). The cornea with a 3‐mm scleral rim was then surgically dissected from the globe and placed into a sample holder (Fig. 2). The natural curvature of the cornea was maintained by clamping the scleral rim within the sample holder and injecting silicon oil (Dow Corning 200/5cS, BDH Laboratory Supplies, Poole, UK) into the chamber behind it. Silicon oil was also injected into the front chamber of the holder so as to maintain a uniform refractive index and reduce light scatter (Kostyuk et al. 2002). The sample holder was then positioned into a specially adapted PYE Unicam (Cambridge, UK) SP8‐100 UV/VIS spectrophotometer with a 1‐mm circular beam, in such a way that light passed through the centre of the cornea in the anterior–posterior direction. A transmission spectrum was measured for each cornea at 10‐nm intervals within the range of 400–700 nm and in the case of the epithelium‐intact control and treated corneas (groups 2, 3, 4, 5, 6, 7 and 8), a second spectrum and corneal thickness measurement was recorded from each specimen following epithelial removal. A further transmission spectrum was obtained for riboflavin solution alone.

As the process of treating and collecting data from forty corneas took 12 hr to complete and progressive swelling of the cornea occurs with increasing time post‐mortem, it was necessary to avoid hydration bias between treatment groups. This was ensured by treating and examining the corneas in five batches, with each batch containing a single cornea from each treatment group.

To normalize the corneal data during the analysis process, baseline readings from the sample holder filled only with silicon oil were recorded over the same wavelength range (400–700 nm) before each corneal measurement.

### Spectrophotometric data analysis

**Figure 3:**
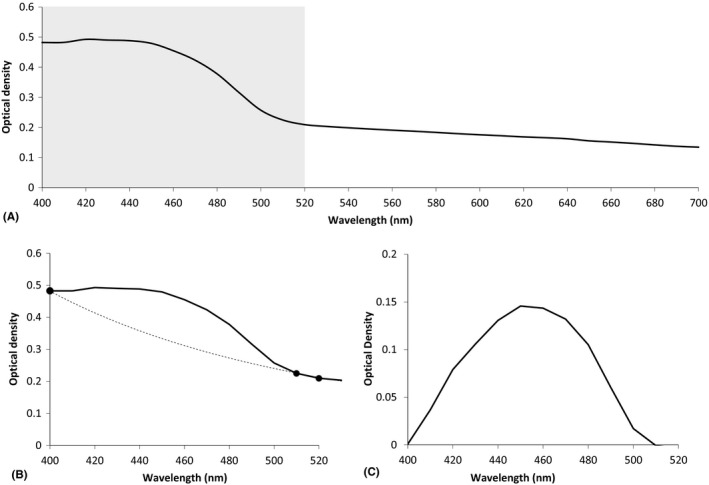
An average optical density spectrum from riboflavin‐treated corneas. (A) The region of absorption due to riboflavin (between 400 and 520 nm) is highlighted in grey. By isolating the riboflavin peak and fitting and subtracting a power law background (dashed line) arising from the optical density of the cornea itself (B), the absorption due to the presence of riboflavin within the tissue could be assessed (C) and compared between treatment groups.

As we have previously shown (Hayes et al. 2008), one of the absorption peaks of riboflavin is evident at 400–520 nm (Fig. 3A). To evaluate the relative amount of riboflavin within the cornea following each treatment, the light transmission data for each cornea were first normalized against its baseline reading. This was performed by expressing the light transmission measurements as a ratio of the transmission recorded from each cornea (mounted in a silicon oil filled chamber) to the baseline reading obtained from the same chamber filled only with silicon oil. The normalized light transmission measurements for individual corneas were then converted into absorbance (optical density) values, which were averaged together for each treatment group and plotted against wavelength. The corneal data were then isolated between 400 and 520 nm to facilitate the detection of differences in riboflavin absorption between individual corneas (before and after epithelial removal) and between corneas in different treatment groups. In order to further isolate the absorption caused by riboflavin, it was necessary to remove a background of scattering and absorption arising from the cornea itself. This was performed by selecting points on either side of the riboflavin peak and then fitting (Fig. 3B) and removing a power law background (Fig. 3C).

### Statistical analysis

As a means of making direct comparisons between treatments groups (in terms of riboflavin uptake), the absorption at 450 nm (in the middle of the riboflavin peak) was examined statistically by means of anova and *post hoc* least significant difference testing.

## Results

### Corneal thickness

**Figure 4:**
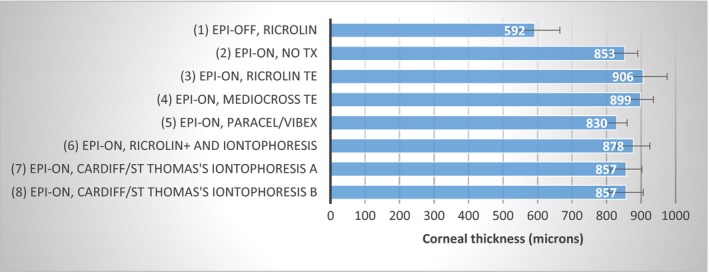
Post‐treatment corneal thickness (±SD).

Figure 4 shows the average post‐treatment corneal thickness for each group. The epi‐off, Ricrolin‐treated corneas (group 1) were significantly thinner (by approximately 280 μm) than all the epithelium‐intact treated (groups 3–8) and untreated (group 2) corneas (p < 0.001). The average thickness of the trans‐epithelial treated corneas in groups 3–5 and the trans‐epithelial‐ and iontophoresis‐treated corneas in groups in 6–8 did not differ significantly from that of the epithelium‐intact, untreated corneas in group 2, which had an average thickness of 853 μm.

### Optical density: evaluation of stromal riboflavin uptake

**Figure 5:**
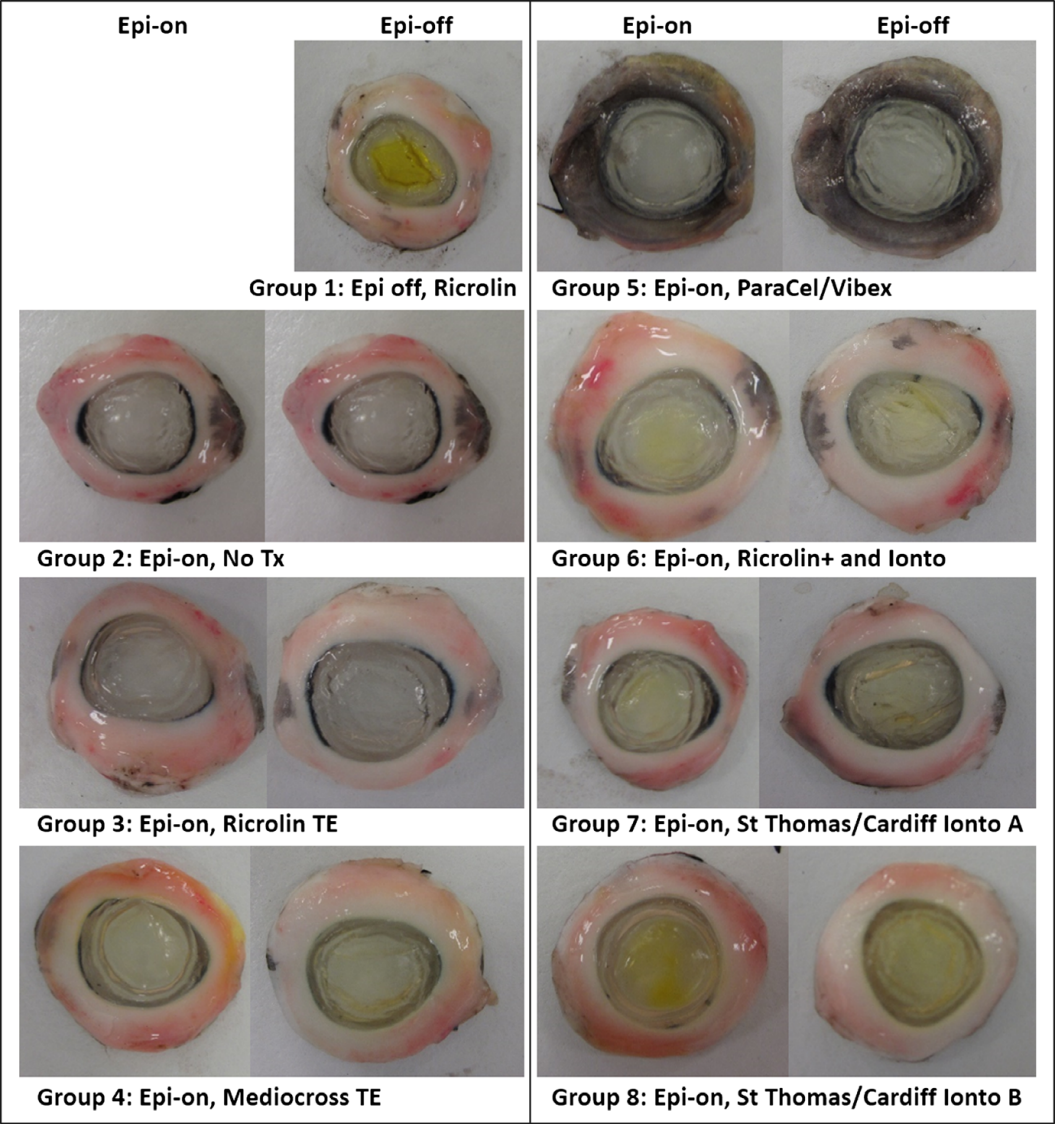
Photographs of treated and untreated corneas captured before (Epi‐on) and after (Epi‐off) epithelium removal. All treatment abbreviations are detailed in Table 1.

Figure 5 shows photographs of each treated and untreated cornea, recorded immediately post‐treatment and again after epithelial removal. The distinctive yellow coloration of riboflavin was most evident in the epithelium‐off treated corneas (group 1) and the epithelium‐intact, St Thomas’/Cardiff Ionto B‐treated corneas (group 8). In contrast, the coloration of corneas treated trans‐epithelially with either Ricrolin TE (group 3) or ParaCel/Vibex (group 5) was similar to that of the untreated corneas (group 2), thus indicating little or no penetration of riboflavin into either the epithelium or stroma. Corneas treated with Mediocross TE (group 4), Ricrolin^+^ and iontophoresis (group 6) and the St Thomas’/Cardiff Iontophoresis protocol A (group 7) showed some evidence of riboflavin penetration immediately post‐treatment, but the yellow coloration (indicating the presence of riboflavin) appeared to diminish after the removal of the epithelium.

**Figure 6:**
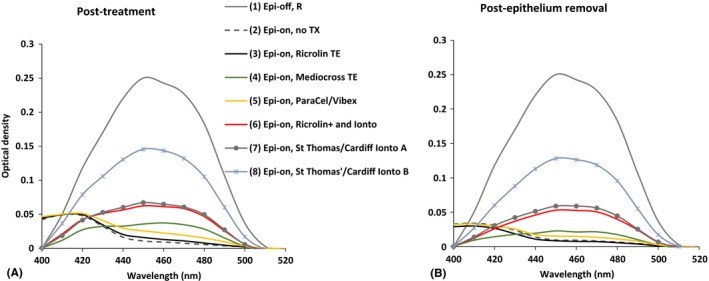
The averaged optical density of untreated corneas (epi‐on, no Tx) and corneas treated with a range of riboflavin delivery protocols (as detailed in Table 1) are shown immediately post‐treatment (A) and after post‐treatment removal of the epithelium (B).

Figure 6A shows the post‐treatment variation in the optical density spectra of corneas treated with different riboflavin delivery protocols alongside the optical density spectra obtained from untreated corneas. Although the riboflavin peak (between 400 and 520 nm) is the most obvious feature in Fig. 5, an additional peak is evident at around 400–420 nm. This peak may correspond to cytochrome *C* absorption (Kostyuk et al. 2002) and could therefore be an indication of cell death. In treatments which have resulted in significant riboflavin uptake (groups 1, 4, 6–8), this additional peak is less evident, presumably because the riboflavin peak masks the effect of cytochrome *C*.

As a means of making direct comparisons between treatment groups (in terms of riboflavin uptake), the absorption at 450 nm was examined post‐treatment and following removal of the epithelium (Table 2 and Fig. 6). As would be expected, the optical density was lowest in the epithelium‐intact, untreated corneas (group 2) (as no riboflavin was applied) and greatest in the epithelium‐removed, riboflavin‐treated corneas (as riboflavin was absorbed directly into the corneal stroma). The optical density in the epi‐off, riboflavin‐treated corneas (group 1) was significantly higher than that of all other untreated (group 2), trans‐epithelial (groups 3–5)‐ and iontophoresis (groups 6–8)‐treated corneas (p < 0.01).

**Table 2:** The effect of treatment and de‐epithelialization on the average (±SD) optical density at 450 nm

	Optical density	
	Post‐treatment	Postepithelium removal
(1) Epi‐off, Ricrolin	0.272 ± 0.087^a^	0.272 ± 0.087^f^
(2) Epi‐on, no Tx	0.011 ± 0.003^b,c^	0.009 ± 0.002^g,h^
(3) Epi‐on, Ricrolin TE	0.015 ± 0.009^b,e^	0.006 ± 0.01^g,j^
(4) Epi‐on, Mediocross TE	0.039 ± 0.029^b,e^	0.023 ± 0.01^g,j^
(5) Epi‐on, ParaCel/Vibex	0.026 ± 0.01^b,e^	0.016 ± 0.016^g,j^
(6) Epi‐on, Ricrolin^+^ and Ionto	0.067 ± 0.046^b,e^	0.056 ± 0.045^g,j^
(7) Epi‐on, St Thomas’/Cardiff Ionto A	0.067 ± 0.038^b,e^	0.061 ± 0.035^g,j^
(8) Epi‐on, St Thomas’/Cardiff Ionto B	0.149 ± 0.066^b,d^	0.135 ± 0.063^g,i^

Statistical differences between epithelium‐intact and epithelium‐removed treated and untreated corneas: a,b and f–g: p < 0.001; c,d and h,i: p < 0.01; and c–e and h–j showed no significant difference.

The optical density of the Ricrolin TE (group 3)‐, Mediocross TE (group 4)‐ and ParaCel/Vibex (group 5)‐treated corneas did not differ significantly from that of the untreated corneas (group 2) before or after epithelial removal, indicating that these protocols resulted in minimal uptake of riboflavin into either the epithelium or the stroma. Although not statistically significant, corneas treated with Ricrolin^+^ + ionto (group 6) and St Thomas’/Cardiff Ionto protocol A (group 7) tended to have a higher optical density than the untreated and non‐iontophoresis‐assisted trans‐epithelial treated corneas.

The St Thomas’/Cardiff‐modified iontophoresis protocol B (group 8) appeared to be the only effective epithelium‐intact method of delivering riboflavin to the corneal stroma as, unlike all the other trans‐epithelial treatments tested, it resulted in significantly higher optical density values at 450 nm than the untreated corneas (p < 0.01). The optical density of the St Thomas’/Cardiff‐modified iontophoresis protocol B‐treated corneas was higher than the untreated corneas when measured immediately post‐treatment (p < 0.01) and similarly, when measured after removal of the epithelium (p < 0.01), thus indicating the successful penetration of riboflavin across the epithelial barrier into the stroma.

## Discussion

Adequate stromal riboflavin uptake is essential to the CXL process as it acts as a photosensitizer to trigger cross‐link formation during UVA exposure and increases UVA absorption within the cornea, affording protection to internal ocular structures, such as the endothelium, lens and retina (Wollensak et al. 2003; Spoerl et al. 2007). In this study, spectrophotometry was used to assess stromal riboflavin uptake and compare the effectiveness of the standard, epithelium‐off delivery technique to that of new and existing trans‐epithelial techniques.

The thickness of corneas treated with trans‐epithelial riboflavin solutions, either with or without iontophoresis, did not differ from that of epithelium‐intact untreated corneas, but the de‐epithelialized corneas treated with the standard hyperosmolar riboflavin solution containing 20% dextran were found to be significantly thinner. Although the removal of the epithelium may account for up to 35% of this reduction in corneal thickness, based on the porcine epithelium measuring approximately 90 μm, the remainder may be attributed to the presence of dextran which creates a high osmotic pressure gradient that drives water from the cornea (Hamaoui et al. 2001). A decrease in corneal thickness following application of similar hyperosmolar riboflavin solutions has been seen previously *in vitro* in porcine corneas (Kling et al. 2010) and clinically in humans (Kymionis et al. 2009). Furthermore, 15% and 20% dextran solutions are known to effectively dehydrate de‐epithelialized donor eye bank corneas to physiological thickness (Hamaoui et al. 2001).

Although Ricrolin TE contains 15% dextran, it was found to have no significant effect on corneal thickness, likely as a consequence of the limited penetration of large dextran molecules across an intact corneal epithelium (Huang et al. 1989; Hayes et al. 2008; Wollensak & Iomdina 2009). This idea was supported by the fact that the optical density in our Ricrolin TE‐treated corneas did not differ significantly from that of the untreated corneas, indicating that there was minimal uptake of riboflavin, and therefore dextran, into either the epithelium or the stroma. Published clinical studies also indicate limited stromal riboflavin penetration in eyes treated with Ricrolin TE in the presence of an intact epithelium; the treatment has been shown to result in a much shallower ‘demarcation line’ (occurring at depths of ~100 μm), than seen following the standard epithelium‐off CXL, where the demarcation line reaches 330 μm (Filippello et al. 2012). It has been postulated that this ‘demarcation line’, which is clearly seen 2–6 weeks after CXL on slit‐lamp biomicroscopy and optical coherence tomography, represents the effective depth of cross‐linking (Seiler & Hafezi 2006; Kymionis et al. 2014), although more research is needed to ascertain its true relationship to the actual CXL process.

The clinical results obtained following trans‐epithelial CXL with Ricolin TE (Sooft Italia trans‐epithelial CXL protocol) are varied. One study of 20 patients reported a cessation in keratoconus progression and an improvement in topographic and visual parameters at 18‐month follow‐up (Filippello et al. 2012). However, another similar sized study with 24‐month follow‐up showed limited stabilization of keratoconus after 12 months, with 50% of cases requiring epithelium‐off retreatment due to progression (Caporossi et al. 2012). In a recently published randomized controlled study, comparing Ricrolin TE trans‐epithelial CXL with the standard epithelium‐off technique, whilst safety with this trans‐epithelial protocol was excellent, 23% of eyes showed evidence of progression at 12 months, compared to stabilization in all eyes treated with standard epithelium‐off CXL (Soeters et al. 2015). Such findings suggest limited efficacy with this trans‐epithelial protocol, which is likely to be a result of limited riboflavin stromal absorption.

Similar to the Sooft Italia trans‐epithelial CXL protocol (Ricrolin TE), we found that the Peschke trans‐epithelial protocol (Mediocross TE) and the Avedro trans‐epithelial protocol (Paracel/Vibex) also resulted in minimal epithelial and stromal riboflavin uptake. This suggests that neither the removal of dextran from the riboflavin formulation nor the addition of Tris, EDTA, sodium chloride and BACS are sufficient to significantly enhance the penetration of riboflavin across an intact epithelium. In accordance with this, a recent study examining epithelial integrity following various trans‐epithelial protocols found Ricrolin TE and Paracel/Vibex to result in the least disruption to the corneal epithelium (Taneri et al. 2014). Whilst to date there have been no published clinical trials examining the effectiveness of the Mediocross or Paracel/Vibex protocols, on the basis of our results and published studies using Ricrolin TE, we suspect that efficacy will be limited compared to the standard epithelium‐off technique.

Although not statistically significant, corneas treated with the Sooft iontophoresis‐assisted delivery protocol (Ricrolin^+^ and ionto) and the St Thomas’/Cardiff‐modified iontophoresis‐assisted protocol A tended to have higher optical density values than untreated and non‐iontophoresis‐assisted trans‐epithelial treated corneas. This is consistent with the findings of Mastropasqua et al. (2014); using human cadaver eyes and high‐performance liquid chromatography, they showed that trans‐epithelial iontophoresis‐assisted delivery resulted in a greater and deeper riboflavin saturation than Ricrolin TE treatment alone but could not match the standard epi‐off technique in terms of stromal riboflavin concentration. Furthermore, clinical examination of 30 patients’ eyes revealed the presence of a significantly shallower and less visible demarcation line in trans‐epithelial treated corneas (Ricolin^+^ and iontophoresis) than in standard epithelium‐off cross‐linked corneas (Bouheraoua et al. 2014).

In contrast to all other trans‐epithelial riboflavin delivery protocols tested in this study, the St Thomas’/Cardiff Iontophoresis protocol B resulted in significant stromal penetration. However, when comparing the effectiveness of the trans‐epithelial treatments, one must consider the riboflavin application time. In the present study, it should be noted that the St Thomas’/Cardiff Iontophoresis protocol B, which involves two 5‐min iontophoresis‐assisted deliveries of Ricrolin^+^ with a 15‐min soakage time in between, has a similar riboflavin application time to that of the Ricrolin TE, Mediocross TE and St Thomas’/Cardiff Iontophoresis protocol A treatments but lasts three times longer than the Paracel/Vibex treatment and six times longer than the Ricrolin^+^ and ionto treatment. Although it cannot be determined from this study whether the incorporation of a longer soakage time in the Paracel/Vibex and Ricrolin^+^ and ionto treatments would significantly improve stromal riboflavin penetration, the results do indicate that a longer iontophoresis time as well as a longer soakage time results in more efficacious trans‐epithelial stromal riboflavin absorption. Further studies are needed to determine the optimum period of riboflavin soakage time and iontophoretic dosage for trans‐epithelial riboflavin delivery, but clearly a modification of the iontophoretic trans‐epithelial technique offers the most promise for epithelium‐on CXL.

Although the St Thomas’/Cardiff protocol B was the most effective trans‐epithelial delivery protocol tested, it was significantly less effective at delivering riboflavin to the stroma than the standard epithelium‐off method. However, it should be noted that the effectiveness of the 2‐min saline rinse (used following trans‐epithelial treatments) as a means of removing riboflavin from the intact epithelium has not yet been validated, and it is possible that the saline rinse may itself cause a loss or dilution of riboflavin within the anterior stroma. As stromal riboflavin uptake and oxygen diffusion are hindered by the presence of an intact epithelium and the epithelium itself (along with any riboflavin that might be contained within it) will absorb UVA light, it is likely that the efficacy of trans‐epithelial CXL will be less than that of the epi‐off technique. However, it is not yet clear exactly what stromal concentration of riboflavin is needed to ensure sufficient cross‐linking for corneal stabilization.

Based on the results presented in this study and the potential patient benefits of trans‐epithelial CXL over the standard epithelium‐off technique in terms of patient comfort, speed of visual recovery and safety, the St Thomas’/Cardiff Iontophoresis protocol B appears to be a promising new alternative to the standard treatment. Furthermore, as the thickness of the epithelium in human corneas is less than that of the porcine cornea (~50 μm (Reinstein et al. 2008) and 90 μm, respectively), the stromal penetration with our modified iontophoresis protocol may be even greater in practice than shown here experimentally. Comparative clinical studies are warranted to assess the efficacy of this protocol on keratoconus stabilization.
